# Antioxidant nitroxides protect hepatic cells from oxidative stress-induced cell death

**DOI:** 10.3164/jcbn.17-60

**Published:** 2018-02-07

**Authors:** Saki Shinto, Yuta Matsuoka, Mayumi Yamato, Ken-ichi Yamada

**Affiliations:** 1 Physical Chemistry for Life Science Laboratory, Faculty of Pharmaceutical Sciences, Kyushu University, 3-1-1 Maidashi Higashi-ku, Fukuoka 812-8582, Japan

**Keywords:** nitroxides, ROS, cell death, oxidative stress, hepatocyte

## Abstract

Oxidative stress causes cell death and induces many kinds of disease, including liver disease. Nitroxides are known to react catalytically with free radicals. In this study, the cell protective activities of nitroxides were compared with those of other antioxidants. Nitroxides showed much greater inhibition of hydrogen peroxide-induced cell death than other antioxidants in a hepatic cell line and in primary hepatocytes. The intracellular oxidative stress level at 24 h after hydrogen peroxide stimulation was significantly decreased by nitroxides, but not by other antioxidants. To clarify the mechanism of cell protection by nitroxides, we investigated whether nitroxides inhibited DNA damage and mitogen-activated protein kinase pathway activation. We found that nitroxides reduced caspase-3 activation and may have ultimately inhibited cell death. In conclusion, nitroxides are very useful for attenuating cell damage due to oxidative stress. Nitroxides are thus a potential therapeutic agent for oxidative stress-related diseases.

## Introduction

In a biological system, reactive oxygen species (ROS) such as superoxide, hydrogen peroxide (H_2_O_2_) and the hydroxyl radical (^•^OH) are continuously generated.^(1)^ They are normally eliminated by host defense systems. However, under certain kinds of stress such as obesity, smoking and radiation exposure, excess amounts of ROS accumulate. ROS damage DNA, lipids and proteins, modify intracellular signaling and ultimately induce cell death.^(2)^ Cell death not only leads to loss of normal cell function, but also accelerates further cell responses such as inflammation. Oxidative stress-induced cell death is often observed in many types of disease.^(3–6)^ The liver is the main site of ROS generation, which occurs through activation of mitochondrial and metabolizing enzymes.^(7)^

Antioxidants scavenge ROS and inhibit oxidative stress-induced cell death. Polyphenols derived from *Silybum marianum* protected mouse liver from carbon tetrachloride-induced damage.^(8)^ Flavonoids extracted from *Morella rubra* inhibited alcohol liver disease in a mouse model.^(9)^ However, to induce such benefits large amounts of antioxidants are needed,^(10,11)^ which can lead to adverse effects. Therefore, it is necessary to identify effective compounds with high anti-oxidative activity. Nitroxides are organic spin compounds with a stable unpaired electron. They have been widely used in many applications, for example as spin labeling agents, synthetic catalysts and contrast agents in MRI scanning.^(12,13)^ Nitroxides react with many types of species, including ROS^(14)^ and organic radicals such as lipid radicals generated by lipid oxidation.^(15,16)^ Importantly, nitroxides can react catalytically with oxidative species. Among many reported nitroxide structures, 4-hydroxy-2,2,6,6-tetramethylpiperidyl-1-oxyl (tempol) is the most widely used experimentally because of its high antioxidative activity and low toxic effects. Tempol reduces pathogenesis in animal models of diseases such as obesity,^(17)^ high blood-pressure,^(18)^ and liver disease.^(19)^ Furthermore, it has been shown in a clinical trial to protect against skin damage during brain cancer radiation therapy.^(20)^ Modifying the substituent at the 4-position in the piperidine ring of tempol changes its reactivity. Some nitroxides, such as 4-amino-2,2,6,6-tetramethylpiperidyl-1-oxyl (tempamine), have higher anti-oxidative activity than tempol,^(21)^ but few studies have examined such compounds. Moreover, the mechanism by which nitroxides protect cells is still unknown, and few reports suggest that they have higher protective ability than other antioxidants such as polyphenols.

In this study, we aimed to investigate whether the nitroxides tempol and tempamine can be used to inhibit cell death, and to compare the cell protective effects of tempol and tempamine with those of other antioxidants. Additionally, we elucidate the mechanism of their protective action in hepatocytes.

## Materials and Methods

### Apparatus and chemicals

Materials were purchased from commercial suppliers and used without further purification. Tempol, tempamine, tiron and EGCG was purchased from Sigma-Aldrich Co. LLC. (St. Louis, MO), Tokyo Chemical Industry Co., Ltd., (Tokyo, Japan), DOJINDO LABORATORIES (Kumamoto, Japan), and Wako Pure Chemical Industries, Ltd. (Osaka, Japan), respectively. All solvents were purified prior to use. Fluorescence and absorbance measurements were recorded on an EnSpire Multimode Plate Reader (PerkinElmer Japan Co., Ltd., Kanagawa, Japan). Chemiluminescence measurements were recorded on an ImageQuant LAS 4000 mini (GE Healthcare, Tokyo, Japan) and a FlexStation 3 (Molecular Devices, Sunnyvale, CA).

### Cell culture

Hepa 1-6 cells (RIKEN BioResource Center, Tsukuba, Japan) were cultured in Dulbecco’s modified Eagle’s medium containing 10% fetal bovine serum (FBS), 1% penicillin-streptomycin solution, and 1% (v/v) Minimal Essential Medium Non-Essential Amino Acids Solution (Nacalai Tesque, Kyoto, Japan) at 37°C in 5% CO_2_.

### Animal experiments

All animal procedures were approved by the Committee on Ethics of Animal Experiments of the Graduate School of Pharmaceutical Sciences, Kyushu University and were conducted according to the Guidelines for Animal Experiments of the Graduate School of Pharmaceutical Sciences, Kyushu University. Seven-week-old male C57BL/6 mice were purchased from Clea Japan, Inc. (Tokyo, Japan). All mice were housed under a 12 h light-dark cycle (lights on from 7:00 to 19:00) with free access to a standard diet (Clea Japan, Inc.) and water.

### Preparation and culture of primary hepatocytes

 Hepatocytes were prepared by the general perfusion method. Primary hepatocytes from 4-month-old C57BL/6N mice (Clea Japan, Inc.) were obtained by collagenase (Wako Pure Chemical Industries, Ltd.) perfusion. The cells were cultured in William’s Medium E (Thermo Fisher Scientific, Inc., MA) containing 10% (v/v) FBS (Thermo Fisher Scientific, Inc.) and 1% penicillin-streptomycin mixed solution (Wako Pure Chemical Industries, Ltd.). The medium was changed after 4 h of culture and the primary hepatocytes were subjected to MTT assays.

### MTT assay

Hepa 1-6 cells or primary hepatocytes were seeded onto 96-well plates at 1 × 10^4^ cells/well and cultured for 24 h. Antioxidants were diluted in PBS containing 1% DMSO and added 30 min before H_2_O_2_ addition. One hour after addition of H_2_O_2_, the medium was discarded and fresh medium was added, and the cells were cultured for a further 23 h. MTT solution 0.5 mg/ml [0.5% (v/v) DMSO] was then added. After incubation for 4 h, the supernatant was removed and DMSO was added. Absorbance at 630 nm was measured using an EnSpire Multimode Plate Reader.

### Intracellular oxidative stress levels at 1 h after H_2_O_2_ stimulation

Hepa 1-6 cells were seeded onto 96-well plates at 1 × 10^5^ cells/well and cultured for 24 h. DCFH-DA solution (2',7'-dichloro-dihydro-fluorescein diacetate, 10 µM) was added 30 min before antioxidants addition with fresh medium. After 30 min H_2_O_2_ was added. One hour after addition of H_2_O_2_, the medium was replaced and the fluorescence intensity was measured using an EnSpire Multimode Plate Reader (excitation 485 nm, emission 535 nm). The supernatant was removed and the same volume of 1 N NaOH in PBS containing 1% (w/v) dodecyltrimethylammonium bromide (DTAB) was added with shaking to extract all proteins. The protein level was measured using a Pierce BCA protein assay kit (Thermo Fisher Scientific, Inc.). The fluorescence level was corrected to the protein level.

### Intracellular oxidative stress levels at 24 h after H_2_O_2_ stimulation

Hepa 1-6 cells were seeded onto 96-well plates at 5 × 10^4^ cells/well and cultured for 24 h. Antioxidants were added 30 min before H_2_O_2_ addition. One hour after addition of H_2_O_2_, the medium was replaced with fresh medium and the cells were cultured for a further 23 h. DCFH-DA solution (2',7'-dichloro-dihydro-fluorescein diacetate, 10 µM) was then added, and after 30 min of incubation the fluorescence intensity was measured using an EnSpire Multimode Plate Reader (excitation 485 nm, emission 535 nm). The supernatant was removed and the same volume of 1 N NaOH in PBS containing 1% (w/v) dodecyltrimethylammonium bromide (DTAB) was added with shaking to extract all proteins. The protein level was measured using a Pierce BCA protein assay kit (Thermo Fisher Scientific, Inc.). The fluorescence level was corrected to the protein level.

### Intracellular NAD^+^ level

Hepa 1-6 cells were seeded onto 96-well plates at 1 × 10^5^ cells/well and cultured for 24 h. Antioxidants were added 30 min before H_2_O_2_ addition. One hour after addition of H_2_O_2_, the medium was removed and the cells were washed with PBS. NaOH (1 N) in PBS containing 1% (w/v) DTAB was added at same amount of PBS, then shaked to extract all NADH and proteins. A sample of this solution was mixed with 0.4 N HCl to extract all NAD^+^, then heated at 60°C for 15 min and neutralized with 0.5 M Tris. The NAD^+^ level was measured using an NAD/NADH-Glo assay (Promega KK, Tokyo, Japan). Chemiluminescence measurements were recorded on a FlexStation 3 (Molecular Devices). The protein level was measured using a Pierce BCA protein assay kit. The NAD^+^ level was corrected to the protein level.

### Western blot analysis

Hepa 1-6 cells were seeded onto a 100 mm dish and cultured until confluent. Tempamine (0.5 mM) was added 30 min before H_2_O_2_ addition. One hour after addition of H_2_O_2_, the cells were washed with PBS and sonicated in radioimmunoprecipitation assay lysis buffer (Santa Cruz Biotechnology, Inc., Dallas, TX) to extract all proteins. The lysate was centrifuged, and the supernatant was collected. The protein level in the supernatant was measured using a Pierce BCA protein assay kit. The proteins were mixed with sample buffer [54.1% (w/v) glycerol, 0.05% (w/v) bromophenol blue, 158.9 mM Tris-HCl pH 6.8, 4.76% (w/v) SDS]. Samples were separated on a 10% (w/v) SDS polyacrylamide gel then transferred to polyvinylidene fluoride membrane (Bio-Rad Laboratories, Berkeley, CA). For immunoblotting, the following primary antibodies were used: anti-phospho-SAPK/JNK (Thr183/Tyr185) (81E11) rabbit monoclonal antibody (#4668), anti-SAPK/JNK antibody (#9252), anti-cleaved caspase-3 (Asp175) antibody #9661 (Cell Signaling Technology Japan, KK, Tokyo, Japan) and anti-GAPDH monoclonal antibody (all Medical & Biological Laboratories Co., Ltd., Nagoya, Japan). Horseradish peroxidase-conjugated goat anti-rabbit (1:4,000, R&D Systems, Inc., Minneapolis, MN) or goat anti-mouse (1:4,000, Millipore Co., Billerica, MA) secondary antibody was used. The immunoreactive bands were visualized using EzWestLumi Plus (ATTO Co., Tokyo, Japan). The band intensity was measured using an LAS4000 mini (GE Healthcare Japan).

### Statistics

All data are presented as means ± SD. An unpaired Student’s *t* test or Tukey-Kramer test was used to assess the statistical significance of differences. Statview ver. 5.0 (SAS Institute Inc., Cary, NC) was used for all statistical analyses. The sample numbers used in each experiment are indicated in the figure legends.

## Results

**Fig. 1:**
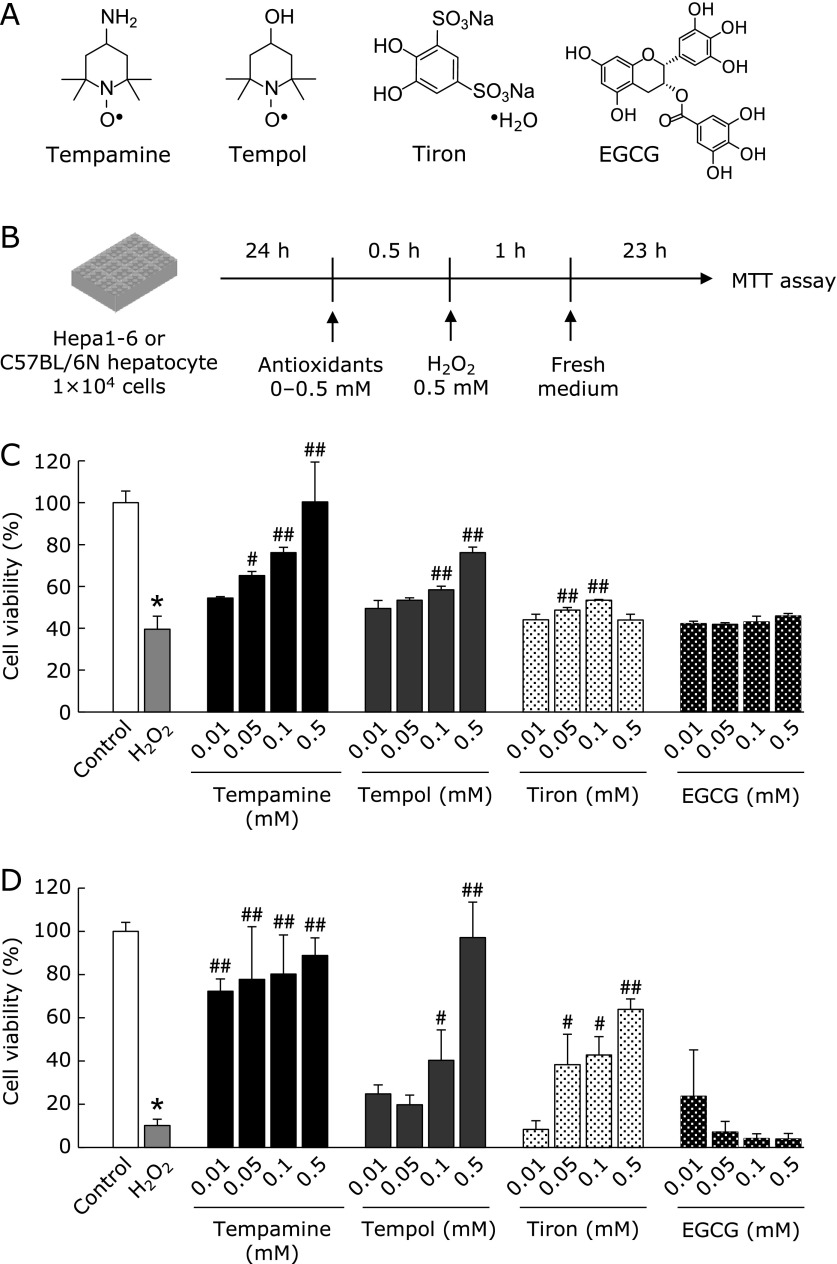
Protective effect of antioxidants against H_2_O_2_-induced cell death. (A) Chemical structure of antioxidants. (B) Experimental design. Hepa 1-6 cells or primary hepatocytes were seeded onto 96-well plates at 1 × 10^4^ cells/well and pre-incubated for 24 h. Antioxidant (0.01, 0.05, 0.1, or 0.5 mM) was added 30 min before H_2_O_2_ (0.5 mM) addition. One hour after addition of H_2_O_2_, the medium was changed and the cells were incubated for a further 23 h. Cell viability was measured by MTT assay. Cell viability in Hepa 1-6 cells (C) and primary hepatocytes (D) are shown. Data are presented as the mean ± SD (*n* = 3) *p<0.01 compared with control. ^#^*p*<0.05, ^##^*p*<0.01 compared with H_2_O_2_.

First, to examine the cell protective effects of nitroxides compared with those of other antioxidants, we used 4,5-dihydroxy-1,3-benzenedisulfonic acid disodium salt (tiron) and (–)-epigallocatechin gallate (EGCG), which are a cell permeable superoxide scavenger and a cell permeable polyphenol, respectively (Fig. 1A and B).^(22,23)^ H_2_O_2_ was used to stimulate oxidative stress in cells of the mouse hepatoma cell line Hepa 1-6 and primary mouse hepatocytes. Cell viability was significantly reduced 24 h after H_2_O_2_ stimulation (Fig. 1C and D). Pretreatment with nitroxides significantly protected against cell death in a dose-dependent manner. Interestingly, the cell protective effects of nitroxides were much higher than those other antioxidants. The same trend was observed in both the cell line and the primary hepatocytes. We therefore used Hepa 1-6 cells for the subsequent experiments.

**Fig. 2:**
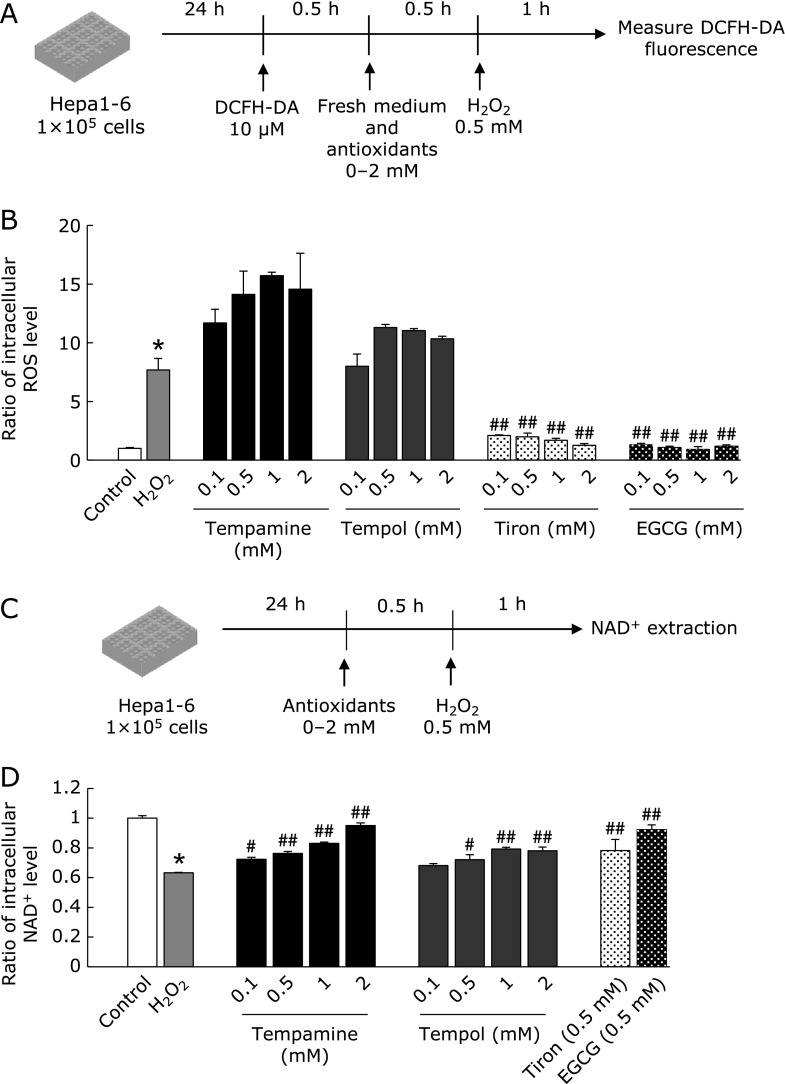
Intracellular oxidative stress and NAD^+^ levels in Hepa 1-6 cells 1 h after H_2_O_2_ stimulation. (A) Experimental design for measuring intracellular ROS levels. Hepa 1-6 cells were seeded onto 96-well plates at 1 × 10^5^ cells/well and pre-incubated for 24 h. The cells were then incubated with DCFH-DA for 30 min, and then the medium was changed. Antioxidant was added 30 min before H_2_O_2_ addition. One hour after addition of H_2_O_2_, intracellular ROS levels were measured via DCFH-DA fluorescence ratio. (B) The ratio of intracellular ROS levels to control group is shown. (C) Experimental design for measuring intracellular NAD^+^ levels. Hepa 1-6 cells were seeded onto 96-well plates at 1 × 10^5^ cells/well and pre-incubated for 24 h. Antioxidant was added 30 min before H_2_O_2_ addition. One hour after addition of H_2_O_2_, intracellular NAD^+^ levels were measured using a commercial kit. (D) The ratio of intracellular NAD^+^ levels to control group is shown. Data are presented as the mean ± SD (*n* = 3) *p<0.01 compared with control. ^#^*p*<0.05, ^##^*p*<0.01 compared with H_2_O_2_.

Next, we hypothesized that the strong protective effect of nitroxides resulted from a catalytic anti-oxidative effect, and that nitroxides exert their anti-oxidative activity for a longer duration than other stoichiometric antioxidants. To clarify this effect, we examined intracellular ROS levels at different times after H_2_O_2_ addition. DCFH-DA, a commercially available fluorescent probe, was used to measure intracellular oxidative stress levels (Fig. 2A). One hour after H_2_O_2_ addition, the fluorescence intensity of DCFH-DA was significantly increased compared with that in vehicle-treated cells (Fig. 2B). Pre-treatment with tiron or EGCG clearly attenuated this increase in fluorescence levels. However, pretreatment with nitroxides was associated with an increase in DCFH-DA fluorescence rather than a decrease.

**Fig. 3:**
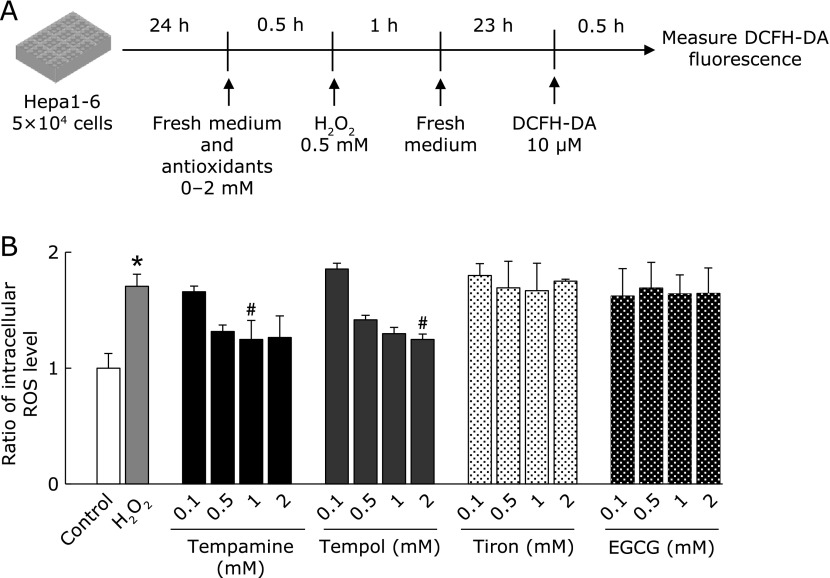
Oxidative stress levels in Hepa 1-6 cells 24 h after H_2_O_2_ stimulation. (A) Experimental design for measuring intracellular ROS levels. Hepa 1-6 cells were seeded onto 96-well plates at 1 × 10^5^ cells/well and pre-incubated for 24 h. DCFH-DA was added and the cells were incubated for 30 min, then the medium was changed. Antioxidant was added 30 min before H_2_O_2_ addition. One hour after addition of H_2_O_2_, the medium was changed and the cells were incubated for a further 23 h. Intracellular ROS levels were measured via DCFH-DA fluorescence. (B) The ratio of intracellular ROS levels to control group is shown. Data are presented as the mean ± SD (*n* = 3) *p<0.01 compared with the control. ^#^*p*<0.05 compared with the H_2_O_2_.

Next, we measured the change in NAD^+^ levels as a marker of DNA damage (Fig. 2C and D). When DNA is damaged, the repair enzyme poly (ADP-ribose) polymerase (PARP) is activated and NAD^+^ is consumed as a substrate.^(24)^ One hour after H_2_O_2_ addition, the NAD^+^ level was significantly reduced (Fig. 2D). Pre-treatment with antioxidants maintained the NAD^+^ level around the control level. Notably, tempamine exhibited a much greater protective effect on NAD^+^ consumption than tempol. Interestingly, 24 h after H_2_O_2_ addition, the DCFH level was reduced only by the nitroxides tempamine and tempol (Fig. 3). These results suggest that the antioxidants used in these conditions significantly inhibited NAD^+^ consumption and may have protected against DNA damage.

**Fig. 4:**
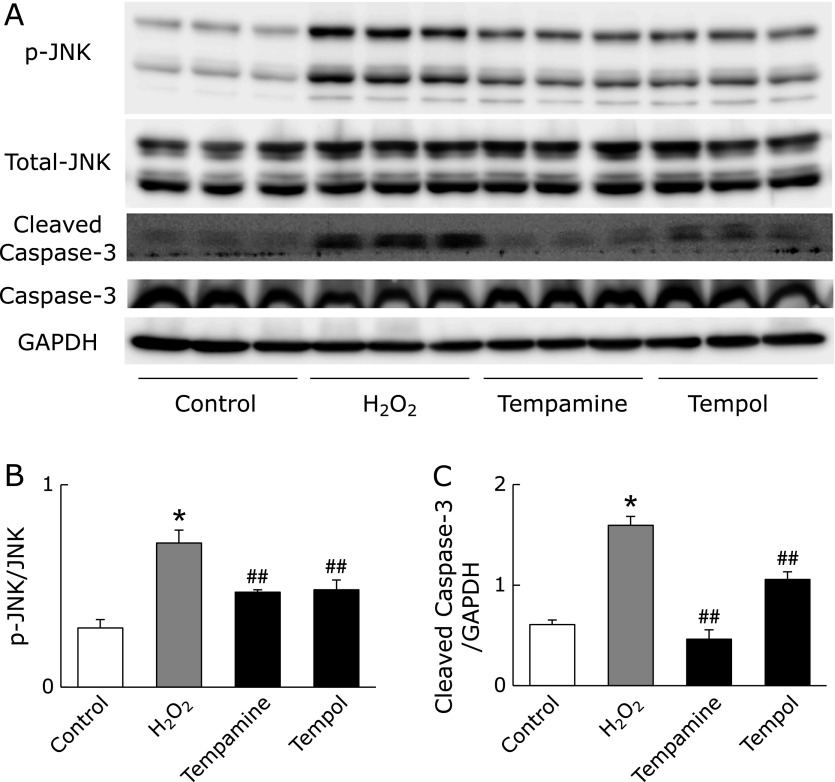
Effect of nitroxides on H_2_O_2_-induced cell death signaling. (A) JNK and caspase-3 activity were evaluated by western blot analysis. p-JNK and total JNK were examined 1 h after H_2_O_2_ stimulation. Cleaved caspase-3 and caspase-3 were examined 24 h after H_2_O_2_ stimulation. Quantification of the band density of p-JNK/JNK (B) and cleaved caspase-3/GAPDH (C) in H_2_O_2_-treated cells. Data are presented as the mean ± SD (*n* = 3) *p<0.01 compared with control. ^#^*p*<0.05, ^##^*p*<0.01 compared with H_2_O_2_.

Next, we further investigated tempamine and tempol, which had great protective effect on cell viability. We hypothesized that tempamine inhibits cell death by protecting against DNA damage. To test this hypothesis, we measured apoptosis signaling. Under strong stress conditions, cell death is induced via the mitogen-activated protein kinase (MAPK) cascade.^(25)^ In particular, c-jun N-terminal kinase (JNK) is activated by DNA damage and activates downstream caspase-3.^(26)^ In this study, 1 h and 24 h of H_2_O_2_ stimulation activated JNK phosphorylation and cleaved caspase-3, respectively (Fig. 4A–C); however, tempamine pretreatment almost completely abolished these signals. Tempol pretreatment also inhibited JNK activation almost completely, but lowered incompletely cleaved caspase-3 level.

## Discussion

In this study, nitroxides exhibited greater protective activity against oxidative stress-induced cell death in hepatocytes than tiron or EGCG (Fig. 1C and D). One hour after H_2_O_2_ addition, intracellular oxidative stress was completely abolished by tiron or EGCG addition (Fig. 2B). However, 24 h after H_2_O_2_ stimulation, only nitroxides inhibited the H_2_O_2_-induced increase in intracellular oxidative stress levels (Fig. 3B). These results suggest that nitroxides can suppress intracellular oxidative stress for a longer duration than other antioxidants. H_2_O_2_ has a longer intracellular half-life than other ROS such as ^•^OH.^(27)^ Therefore, after the medium was changed and extracellular H_2_O_2_ was eliminated, ^•^OH was produced sustainably from residual intracellular H_2_O_2_. Indeed, 24 h after H_2_O_2_ addition, the intracellular oxidative stress level was about 1.7-fold higher than control (Fig. 3B). Stoichiometric antioxidants such as tiron and EGCG can scavenge ROS for a limited time then lose their anti-oxidative capacity, whereas nitroxides can scavenge ROS catalytically. Thus, nitroxides exhibit long-lasting anti-oxidative activity and inhibit intracellular ROS 24 h after H_2_O_2_ addition, and so might offer more effective protection against cell death than tiron or EGCG. At 1 h after H_2_O_2_ stimulation, tempol and tempamine increased DCFH oxidation. This might be because oxoammonium cation form of nitroxide can directly oxidize DCFH (data not shown).

Elevation of intracellular oxidative stress levels damages DNA and induces cell death. We measured the level of NAD^+^ as a marker of DNA damage. Watson *et al.*,^(28)^ reported that H_2_O_2_ treatment induced NAD^+^ reduction via PARP activation. They reported that incubation of PARP inhibitor with H_2_O_2_ led to inhibit 94.2% NAD^+^ reduction. This means that NAD^+^ reduction after H_2_O_2_ treatment almost completely depends on PARP activation, i.e., DNA damage. Thus, we measure NAD^+^ level as the marker of DNA damage. In this study, H_2_O_2_ addition significantly reduced intracellular NAD^+^ levels (Fig. 2D). Although all four antioxidants restored NAD^+^ levels to control levels, tempamine exhibited a higher protective activity than tempol. Tempol has a hydroxyl group at the 4-position of its piperidine ring, whereas tempamine has a primary amine group. The phosphate group of DNA is negatively charged, and tempamine, which is positively charged because of its amine group, has a higher affinity for DNA than tempol. Hence, tempamine might offer greater protection against DNA damage. This finding is in keeping with a previous report.^(26)^ Here, although nitroxides, especially tempamine, might have protected against cell damage by protecting DNA, other protective mechanisms may be involved. Notably, tempamine protected against cell death completely at 0.5 mM (Fig. 1C and D), but under the same conditions, NAD^+^ levels did not recover completely (Fig. 2D). Additionally, tiron or EGCG provided the same degree of protection against NAD^+^ consumption as tempamine at 1 h after H_2_O_2_ addition (Fig. 2D). This is a limitation of this study: mechanisms other than NAD^+^ consumption and PARP activation may exist that protect against DNA damage.

**Fig. 5:**
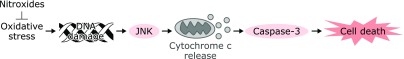
Proposed mechanism of protecting effect of tempamine.

When DNA is damaged, JNK, one of the MAPK proteins, is activated. In this study, JNK phosphorylation levels significantly increased after H_2_O_2_ addition (Fig. 4A and B). Tempamine and tempol partially attenuated this increase in JNK phosphorylation levels. Further downstream in the JNK-related cell death pathway, caspase-3 was activated by H_2_O_2_ stimulation, and tempamine completely inhibited this activation (Fig. 4A and C). This result indicates that H_2_O_2_-induced JNK activation does not represent the complete mechanism of the cell protective effect of nitroxides. Oxidative stress-induced cell death involves many pathways, such as opening of the mitochondrial permeability transition pore.^(29)^ However, tempamine is an effective compound for protecting against H_2_O_2_-induced cell death, and might suppress the most upstream stress (Fig. 5).

In conclusion, possibly because nitroxides reduced oxidative stress catalytically, they had considerably higher anti-oxidative activity and inhibited cell death more effectively than other antioxidants. Nitroxides can reduce oxidative stress for a longer period of time than other antioxidants, meaning that lower doses are necessary to inhibit cell death. These results suggest that nitroxides may emerge as effective drugs to treat and suppress oxidative stress diseases.

## Abbreviations

DCFH-DA: 2',7'-dichloro-dihydro-fluorescein diacetate
DTAB: dodecyltrimethylammonium bromide
EGCG: (−)-epigallocatechin gallate
FBS: fetal bovine serum
H_2_O_2_: hydrogen peroxide
JNK: c-Jun-NH_2_-terminal kinase
MAPK: mitogen-activated protein kinase
O_2_^•−^: superoxide anion radical
^•^OH: hydroxyl radical
PARP: poly (ADP-ribose) polymerase
ROS: reactive oxygen species
tempamine: 4-amino-2,2,6,6-tetramethylpiperidyl 1-oxyl
tempol: 4-hydroxy-2,2,6,6-tetramethylpiperidyl 1-oxyl
tiron: 1,2-dihydroxy-3,5-benzenedisulfonic acid disodium salt
